# Supplementary material for: Evidence supported by Mendelian randomization: impact on inflammatory factors in knee osteoarthritis
Source: Front Med (Lausanne). 2024 May 28;11:1382836. doi: 10.3389/fmed.2024.1382836 (PMC11165061; doi:10.3389/fmed.2024.1382836)
Supplement: Supplementary file 1 [file Data_Sheet_1.ZIP › data/GCST90274827_5e6/827.leaveoneout.pdf]

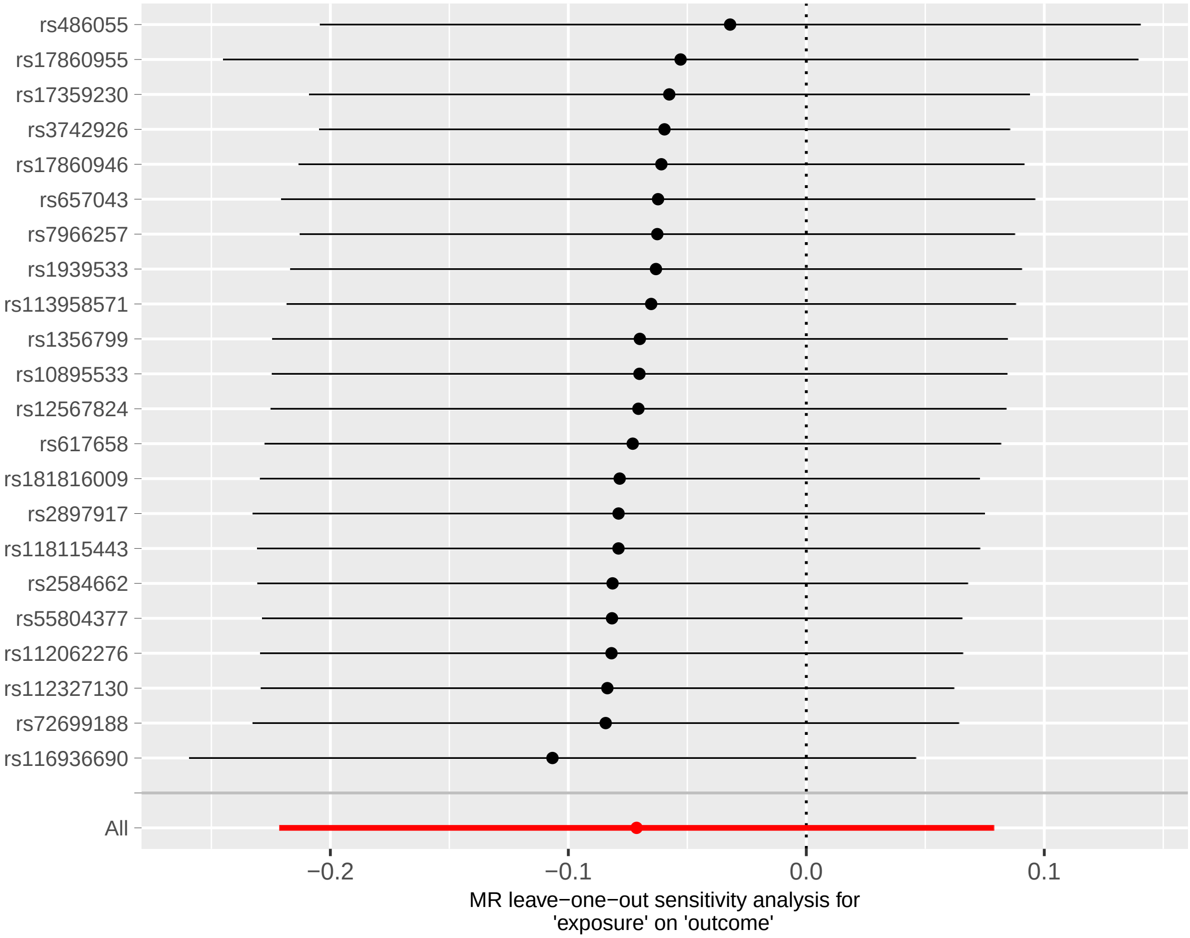
rs486055
rs17860955
rs17359230
rs3742926
rs17860946
rs657043
rs7966257
rs1939533
rs113958571
rs1356799
rs10895533
rs12567824
rs617658
rs181816009
rs2897917
rs118115443
rs2584662
rs55804377
rs112062276
rs112327130
rs72699188
rs116936690
All
−0.2
−0.1
0.0
0.1
MR leave−one−out sensitivity analysis for
'exposure' on 'outcome'
